# Cesarean Section under General Anesthesia over 10 Years at Tertiary Care Center: A Descriptive Cross-sectional Study

**DOI:** 10.31729/jnma.8738

**Published:** 2024-09-30

**Authors:** Smriti Basnet, Prabineshwor Prasad Lekhak, Prabin Subedi, Sushila Lama Moktan

**Affiliations:** 1 Kathmandu Medical College and Teaching Hospital, Sinamangal, Kathmandu, Nepal; 2 Department of Anesthesia and Intensive Care, Kathmandu Medical College and Teaching Hospital, Sinamangal, Kathmandu, Nepal

**Keywords:** *cesarean section*, *emergency*, *general anesthesia*, *gestation*

## Abstract

**Introduction:**

The global surge in cesarean deliveries necessitates safe anesthetic practices to optimize outcomes. While the neuraxial block is the preferred method, specific conditions warrant general anesthesia. This study is aimed to determine the prevalence of cesarean sections under general anesthesia at a tertiary care center.

**Methods:**

A descriptive cross-sectional study was conducted on cesarean sections performed between April 14, 2013, and April 13, 2023, at a tertiary care center. Ethical approval was obtained from the Institutional Review Committee (Reference number: 20092023/01). Total sampling was done. Data for the past ten years were manually collected from hospital records using a self-structured proforma and analyzed using Microsoft Excel 2018 and IBM SPSS version 26. The point estimate and 95% confidence interval were calculated for the study.

**Results:**

In this study, there were 216 (2.98%) (95% CI: 2.59-3.38%) cesarean sections under general anesthesia in 10 years. Notably, among them, there were 103 (47.69%) primigravida, nullipara 130 (60.19%), 135 (62.50%) with no living children, and 161 (74.54%) without any past abortions. In most cases, 182 (84.26%) were emergency procedures and 34 (15.74%) were elective. Fetal bradycardia with distress was the primary indication observed in 121 (56.02%) cases, followed by 32 (14.81%) maternal requests. Additionally, 21 (2.66%) cases were found initially planned as cesarean sections under subarachnoid block but were converted to general anesthesia. The prevalence in this study was found within the recommended limits.

**Conclusions:**

The findings highlight that the majority of these procedures were emergency cases, predominantly due to fetal distress, with a notable proportion involving primigravida and nulliparous women.

## INTRODUCTION

Cesarean section is a surgical procedure that is widely performed across the globe, with rising rates in developing countries surpassing the WHO recommendation rate of 10-15%.^1^ Neuraxial anesthesia is the preferred choice, as the risk of mortality with general anesthesia is 16.7 times higher than with regional anesthesia.^2,3^ Thus, the use of general anesthesia for cesarean section is recommended for less than 5% of elective and less than 15% of emergency cases.^3^

General anesthesia may be warranted in emergencies due to a perceived lack of time for neuraxial block. This leads to general anesthesia being used for maternal, obstetric, and fetal indications, failed technique, or on maternal request.^4^ However, advances in regional anesthesia have reduced general anesthesia reliance globally.^3^

This study aims to fill the gap in the existing literature regarding the prevalence and indications for general anesthesia in cesarean sections at a tertiary care center in Nepal.

Hence, this study aimed to determine the prevalence of cesarean section performed under general anesthesia at a tertiary care center in Nepal.

## METHODS

This descriptive cross-sectional study was conducted on cesarean section under general anesthesia at the Department of Anesthesia and Intensive Care of Kathmandu Medical College Teaching Hospital (KMCTH), Sinamangal, Kathmandu, Nepal, after obtaining ethical clearance from the Institutional Review Committee (Reference number: 20092023/01). The study was conducted between December 1 and December 31, 2023. Total sampling method was employed and all patients who underwent elective and emergency cesarean section from April 14, 2013 to April 13, 2023 were analyzed in the study. Manual counting of the cases that had undergone cesarean section irrespective of anesthesia of choice in 10 years was done only to facilitate calculating the prevalence of cesarean section under general anesthesia.

Any clinical data requiring clarification, difficult to interpret or missing were excluded from the study. The conversion of a failed spinal to general anesthesia were excluded as it is not considered an appropriate indication for the latter procedure. Consequently, this scenario is not factored into the calculation of the indication of cesarean section performed under general anesthesia. The data was carefully collected manually and recorded into a self-structured proforma.

The methodology in a similar research project conducted in Nepal was carefully adapted to our research, ensuring its relevance and accuracy.^5^ The proforma comprised a section for ascertaining crucial demographic parameters, notably the age bracket of patients undergoing cesarean sections under general anesthesia. It also encompassed sections for determining the primary indications necessitating such procedures, assessing the prevalent obstetric indices in this context, identifying the predominant nature of these surgeries, and differentiating between elective and emergency cases.

Data was collected using Microsoft Excel 2018, which was later analyzed using Statistical Package for the Social Sciences (IBM SPSS) version 26.

## RESULTS

There were 7240 cesarean sections performed over the period of 10 years, out of which, 216 (2.98%; 2.593.38, 95% CI) cesarean sections were performed under general anesthesia. Out of total cesarean sections performed, emergency cesarean sections under general anesthesia was 182 (2.51%) and elective cesarean sections under general anesthesia was 34 (0.47%).

The cesarean section under general anesthesia in the year 2014 was 16 (3.21%), in 2015 it was 22 (4.20%) and in the year 2021 it was 11 (1.71%) (Table 1).

**Table 1:** Number of cesarean sections (CS) under general anesthesia (GA) by year (n= 216).

Year^*^	Total number of CS	CS under GA n (%)
April 14, 2013 to April 13, 2014	498	16 (3.21)
April 14, 2014 to April 13, 2015	524	22 (4.20)
April 14, 2015 to April 12, 2016	741	15 (2.02)
April 13, 2016 to April 13, 2017	704	26 (3.69)
April 14, 2017 to April 13, 2018	912	27 (2.96)
April 14, 2018 to April 13, 2019	1019	39 (3.83)
April 14, 2019 to April 12, 2020	839	25 (2.98)
April 13, 2020 to April 13, 2021	571	14 (2.45)
April 14, 2021 to April 13, 2022	643	11 (1.71)
April 14, 2022 to April 13, 2023	789	21 (2.66)
Total	7240	216 (2.98)

* Data from April 14 to April 13 of the next year was taken to match the Nepali calendar year.

The age of patients ranged from 16-47 years with mean age of 27.66±4.98 years. In our study sample, there were 103 (47.69%) primigravida, 130 (60.19%) nullipara, 135 (62.50%) patients with no living children, and 161 (74.54%) without any past abortions (Table 2).

**Table 2:** Cesarean section under general anesthesia on various obstetric groups (n = 216).

Variables	n (%)
**Age Group**	
Below 18 years	1 (0.46)
18 to 24 years	58 (26.86)
25 to 34 years	140 (64.81)
35 to 44 years	16 (7.41)
45 years and above	1 (0.46)
**Gravida**	
1 (Primigravida)	103 (47.69)
2	65 (30.09)
3	36 (16.67)
4	8 (3.70)
5	3 (1.39)
8	1 (0.46)
**Parity**	
0 (Nullipara)	130 (60.19)
1	65 (30.09)
2	17 (7.87)
3	3 (1.39)
5	1 (0.46)
**Number of living children**	
0	135 (62.50)
1	65 (30.09)
2	12 (5.56)
3	3 (1.39)
5	1 (0.46)
**Number of past abortions**	
0	161 (74.54)
1	44 (20.37)
2	9 (4.17)
3	1 (0.46)
4	1 (0.46)

**Figure 1:**
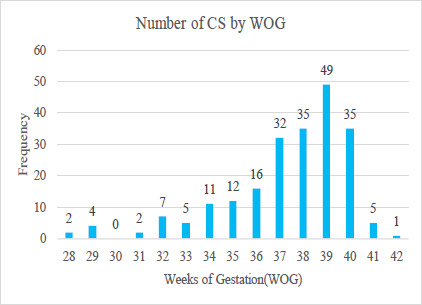
Period of gestation undergoing cesarean section under general anesthesia (n = 216).

Out of the total cesarean sections under general anesthesia, 49 (22.68%) were performed in the 39^th^ week of gestation. The median gestational period for these patients was 38 weeks. The range of gestational weeks for patients undergoing cesarean section under general anesthesia was found to be between 28 and 42 months. The CS under GA at 39 weeks was 49% (Figure 1).

Fetal bradycardia with fetal distress 121 (56.02%), maternal request 32 (14.81%) were causes of cesarean section under general anesthesia (Table 3). It is worth noting that, in 21 (9.72%) cases, it was initially decided to conduct cesarean sections under subarachnoid block. However, these cases were later converted to general anesthesia.

**Table 3:** Indication for cesarean section (n= 216).

Variable	n (%)
Fetal bradycardia	121 (56.02)
On maternal request for CS under GA	32 (14.81)
Failed induction of labor with fetal distress	18 (8.33)
Hepatic abnormalities	11 (5.09)
Eclampsia	10 (4.63)
Cord prolapse	9 (4.17)
Severe pre-eclampsia	8 (3.70)
Antepartum hemorrhage (abruptio placentae, placenta previa)	7 (3.24)

## DISCUSSION

Over ten years, the prevalence of cesarean sections performed under general anesthesia was 216 (2.98%). Comparatively, a similar study conducted in Western Nepal over four years showed a prevalence of 4.84%.^5^ Another study conducted at Western Regional Hospital showed a lower prevalence of 1.87%.^6^ Internationally, there is a wide range of prevalence; studies conducted in Belgium between 2007 and 2015 showed a prevalence of 1.9%, while a tertiary care center in Singapore reported a prevalence of 20.4%.^4,7^ In Boston, USA, the prevalence ranged between 0.5 - 1%.^8^ These variations in prevalence can be attributed to factors such as the duration of data collection, the types of patients encountered, the available resources and services, the experience of the anesthesiologist performing the procedure, hospital protocols, and the demographic patterns of the patients.

The study found that the average age of women undergoing a cesarean section under general anesthesia was 27.66 years, which aligns with other studies where the majority of patients are under the age of 30.^1,6^ The majority of patients in the study were primigravida and nulliparous mothers, which may have contributed to the findings. The median week of gestation in our study was 38 weeks. Studies have shown that elective cesarean deliveries were most commonly performed in the first and second halves of the 37^th^ week of gestation, with rates of 91.0% and 92.6%, respectively.^9^

The majority of cesarean sections under general anesthesia in our study were performed at 39 weeks of gestation. Approximately 60.19% of the women were nulliparous among the women undergoing CS under GA. It is noteworthy that the American College of Obstetricians and Gynecologists (ACOG) recommends postponing these procedures until 39 weeks of gestation to ensure optimal fetal outcomes, particularly for nulliparous women.^10^ This applies to all cesarean sections; under general anesthesia or subarachnoid block. It is also noteworthy that 47.69% of the total sample were primigravida, consistent with a previous study conducted at the same institution with 51%.^1^ Studies have shown that primigravida has a higher incidence of cesarean sections compared to multigravida.^11,12^ A study on the profile of cesarean sections at Kirtipur Hospital, Kathmandu, Nepal shows that the rate of primigravida is as high as 65.9%.^13^ Furthermore, certain studies suggest a higher incidence of obstetric complications such as preterm labor, oligohydramnios, deep transverse arrest, fetal distress, intrauterine growth restriction (IUGR), and placenta previa among primigravida.^12,14^

Out of total cesarean sections performed over the period of 10 years, 182 (2.51%) were performed under general anesthesia in emergency circumstances. Similarly, 34 (0.47%) were performed electively. This latter figure falls within the Royal College of Anesthetists' recommended limits, which suggest that elective cesarean sections should not exceed a prevalence of 5% and emergency cesarean sections should not exceed a prevalence of 15%.^3^ The study's findings are consistent with other research, which demonstrates that general anesthesia is more frequently required in emergency cases than in elective ones.^5,7,8^ However, a study conducted in western Nepal revealed that higher rates of elective cesarean sections were performed under general anesthesia than emergency cesarean sections due to a higher rate of failed spinal anesthesia.^5^ It has been noted that more emergency procedures tend to utilize general anesthesia compared to elective ones. The reason behind this trend is the lack of time for spinal anesthesia, which has been known to have a slower and unpredictable time required for a surgical block to develop to the desired level. This limitation is particularly relevant in emergent conditions such as category-I cesarean sections.^15^ The availability of a Neonatal Intensive Care Unit department, which typically handles high-risk pregnancies and preterm cases, can influence the high rate of emergency cesarean sections performed under general anesthesia at our institution.

The predominant indication for cesarean sections performed under general anesthesia was fetal bradycardia, accounting for 121 (56.02%) of all cases. Meanwhile, maternal request accounted for 32 (14.81%). These findings are consistent with those of a similar study conducted in Belgium.^7^ In contrast, two other studies in Nepal and Singapore found that failed spinal anesthesia and maternal request were the most common indications, respectively.^4,5,6,7^ Fetal distress was detected by monitoring the fetal heart rate using cardiotocography. However, this method has been known to overestimate the degree of fetal discomfort, leading to a higher rate of cesarean sections. While fetal scalp blood pH estimation is considered the gold standard investigation to diagnose fetal distress, it is not currently being implemented in our center. Our institution has a lower rate of failed spinal anesthesia compared to other studies conducted in Nepal (62.85% and 32% respectively), and this may be attributed to the fact that anesthetic techniques are performed mainly by senior residents under the guidance of a team of experienced anesthesiologists at the capital of the country.^5,6^ However, it is worth noting that the rate of failed spinal techniques is still higher than the data from Boston, USA, where it is less than 4%.^8^ The transition from unsuccessful spinal anesthesia to general anesthesia is not deemed a suitable reason for opting for the latter method. As a result, this particular situation is not taken into account when determining the indication of cesarean sections conducted with general anesthesia.

This highlights the need for a multicentric study in Nepal to evaluate the cesarean section rate performed under general anesthesia, its indications, associated factors, and outcomes.

The constraints imposed by a single site of study signify that the conclusions drawn from this research due to limited resources may not be reliably extrapolated or applied to broader populations, as the demographics, characteristics, and circumstances of the participants within this study may not fully represent the diversity and variables present in larger, more heterogeneous populations specifically representing the whole country. The data for this research emanated from manually recorded cases in the register books spanning the last ten years. The exclusion of missing, unclear, or improperly recorded data might have led to an underrepresentation of the total number of cases.

## CONCLUSIONS

The study indicates that cesarean sections under general anesthesia have been relatively less at the tertiary care center over the past decade. Most of these procedures were emergency cases, often due to fetal distress, and involved a significant number of primigravida, nullipara, and women with no prior abortion. Compared to similar studies, a lower prevalence of general anesthesia was observed, and its use for both elective and emergency cesarean sections remains within the recommended threshold.

## References

[1] Prasad A, Bhandari G, Saha R (2017). Profile of Cesarean Section at Kathmandu Medical College.. J Nepal Health Res Counc..

[2] Hawkins JL, Koonin LM, Palmer SK, Gibbs CP (1997). Anesthesia-related Deaths during Obstetric Delivery in the United States, 1979-1990.. Anesthesiology..

[3] Colvin JR (2012). Raising the Standard: A Compendium of Audit Recipes for Continuous Quality Improvement in Anaesthesia.. Royal College of Anaesthetists.

[4] Kan RK, Lew E, Yeo SW, Thomas E (2004). General anesthesia for cesarean section in a Singapore maternity hospital: a retrospective survey.. International Journal of Obstetric Anesthesia..

[5] Adhikari KM, Lakhe G, Adhikari AS (2019). Cesarean Sections under General Anesthesia at a Tertiary Care Center in Western Nepal: A Descriptive Cross-sectional Study.. J Nepal Med Assoc..

[6] Sigdel R, Lama M, Gurung S, Timilsina S (2018). Anesthesia practice in cesarean delivery in tertiary care hospital: a retrospective observational study.. Medical Journal of Pokhara Academy of Health Sciences..

[7] Cools E, de Velde MV (2018). Incidence, causes and complications of general anesthesia for cesarean section: a 9-year retrospective analysis of a large tertiary centre..

[8] Palanisamy A, Mitani AA, Tsen LC (2011). General anesthesia for cesarean delivery at a tertiary care hospital from 2000 to 2005: a retrospective analysis and 10-year update.. International Journal of Obstetric Anesthesia..

[9] Hoshino M, Shinozaki H, Kitahara Y, Kameda T, Hayashi K, Ogawa S (2022). Optimal timing of elective repeat cesarean deliveries of term singleton pregnancies: A multicenter cross-sectional study.. Taiwanese Journal of Obstetrics and Gynecology..

[10] Measure: Cesarean Delivery for Nulliparous (N T S) Women (Appropriate Use)

[11] Kumar R, Chaudhary R, Dhama V, Singh S (2023). Clinical study of primary cesarean section among multigravida in a tertiary care hospital.. Int J Reprod Contracept Obstet Gynecol..

[12] Bamon W, Goswami SN, Roy I, Saikia N A Comparative Study of Primary Caesarean Section in Primigravida and Multigravida in a Tertiary Care Hospital in Shillong, Meghalaya.. SIJOG.

[13] Pradhan P, Shrestha S, Rajbhandari PK, Dangal G (2014). Profile of Caesarean Section in Kirtipur Hospital.. Nepal j obstet gynaecol..

[14] Amin F, Tali TA, Ara R, Amin H (2023). Comparative analysis of pregnancy complications: primigravida versus multigravida.. International Journal of Research in Medical Sciences..

[15] Kathirgamanathan A, Douglas MJ, Tyler J, Saran S, Gunka V, Preston R (2013). Speed of spinal vs general anesthesia for the category-1 caesarean section: a simulation and clinical observation-based study.. Anaesthesia..

